# A Randomised Controlled Trial to Compare the Effect of Ramosetron and Ondansetron in Prevention of Postoperative Nausea and Vomiting in Patients Undergoing Laparoscopic Gynaecological Procedures

**DOI:** 10.7759/cureus.29200

**Published:** 2022-09-15

**Authors:** Sashmita Das, Anil Kumar, Anshu Gupta, Ajai Kumar

**Affiliations:** 1 Anesthesiology and Critical Care, Lady Hardinge Medical College, New Delhi, IND

**Keywords:** incidence of postoperative nausea and vomiting, ramosetron and ondansetron, rescue antiemetics, laparoscopic gynecological procedures, postoperative nausea and vomiting

## Abstract

Background and objective

The antiemetic drug is one of the most common armamentariums in an anaesthesiologist's pharmacopoeia to prevent postoperative nausea and vomiting (PONV). PONV is one of the usual side effects after general anaesthesia, especially in female patients (21%) and after laparoscopic surgery (60%). This study aimed to compare the efficacy of ondansetron with ramosetron.

Methodology

After institutional ethical clearance and informed written consent, one hundred female patients scheduled for laparoscopic gynaecological surgeries were selected for this prospective, double-blinded, randomised interventional study. These patients were further subdivided into two equal groups (50 in groups R and O). Group R received ramosteron 0.3mg, and group O received ondansetron 8mg 30 minutes before the end of surgery. Patients were assessed between 0-2, 2-6, 6-12 and 12-24 hrs in the postoperative period. The primary objective of this study was to compare the effect of a single dose of ramosetron (0.3mg) with a single dose of ondansetron (8mg) for the prevention of PONV after general anaesthesia in laparoscopic surgeries. The secondary goal was to record the time of occurrence of the first episode of PONV, the need for rescue antiemetics, patient satisfaction scores, and to look for any side effects.

Results

This study shows no significant difference in the reduction of PONV incidence between group O and group R in the first 24 hours of the postoperative period. The overall incidence of PONV was significantly higher in the early postoperative (0-6 hrs) than in the late postoperative period (6-24 hrs), i.e., 51% and 13%, respectively. The requirement of rescue antiemetic was higher in group O than in group R but not statistically significant. In our study, both groups had similar patient satisfaction scores. Headache was the most common side effect and was noted in 9% of the patient population.

Conclusion

We conclude that ramosetron is as effective as ondansetron in preventing the incidence and severity of PONV up to 24 hours postoperatively.

## Introduction

Postoperative nausea and vomiting (PONV) are ubiquitous and distressing symptoms that prolong the hospital stay and reduce patient satisfaction after surgery. The incidence of PONV varies from 20% to 30% in female patients to 70%-80% in patients at high risk (non-smokers, female patients, history of motion sickness and use of postoperative opioids) [1,2]. Laparoscopy further increases the incidence of PONV to 56%-93% [3]. Amongst the different explanations are high intra-abdominal pressure, rapid peritoneal distention, stretching of the peritoneum due to insufflation and diffusion of carbon dioxide into the bowel leading to bowel distention [4]. PONV is often associated with increased morbidity due to pulmonary aspiration of gastric contents and fluid-electrolyte imbalance, leading to dehydration and delayed hospital discharges in surgical patients [5]. Anaesthesiologists should implement effective evidence-based measures to prevent PONV in routine practices [6].

Despite the progress over the past few decades with the advent of better anaesthetics and antiemetic agents, the optimal approach for managing PONV is not straightforward and is still a challenge. For the prevention of PONV, selective serotonin 5-hydroxy tryptamine type 3 (5-HT3) receptor antagonists are considered one of the first-line therapy because of their efficacy and fewer side effects compared to other antiemetics [7].

Ondansetron has been the most frequently used and researched antiemetic drug to prevent PONV. Ramosetron is a comparatively recent antiemetic having a selective 5-HT3 receptor antagonist. It exhibits significantly greater binding affinity for the 5-HT3 receptor with a slower dissociation rate from the receptor binding site, resulting in more potent and prolonged receptor antagonising effects than other older 5-HT3 receptor antagonists [8,9]. In addition to this, the elimination half-life of ramosetron is longer (9hrs) than that of ondansetron (3.5hrs) [8,10].

However, available literature compares ramosetron and ondansetron to prevent PONV, but the results are variable. Some studies found no significant differences between the two, while others show ramosetron is a better agent [11-13]. Therefore, a randomised, double-blind study was designed to evaluate the effect of ramosetron on the prevention of PONV compared to the effect of ondansetron in patients undergoing laparoscopic gynaecological procedures. We hypothesised that by virtue of 5-HT3 blockers, ondansetron and ramosetron are equally effective in preventing PONV in patients undergoing laparoscopic gynaecological procedures.

## Materials and methods

**Figure 1:**
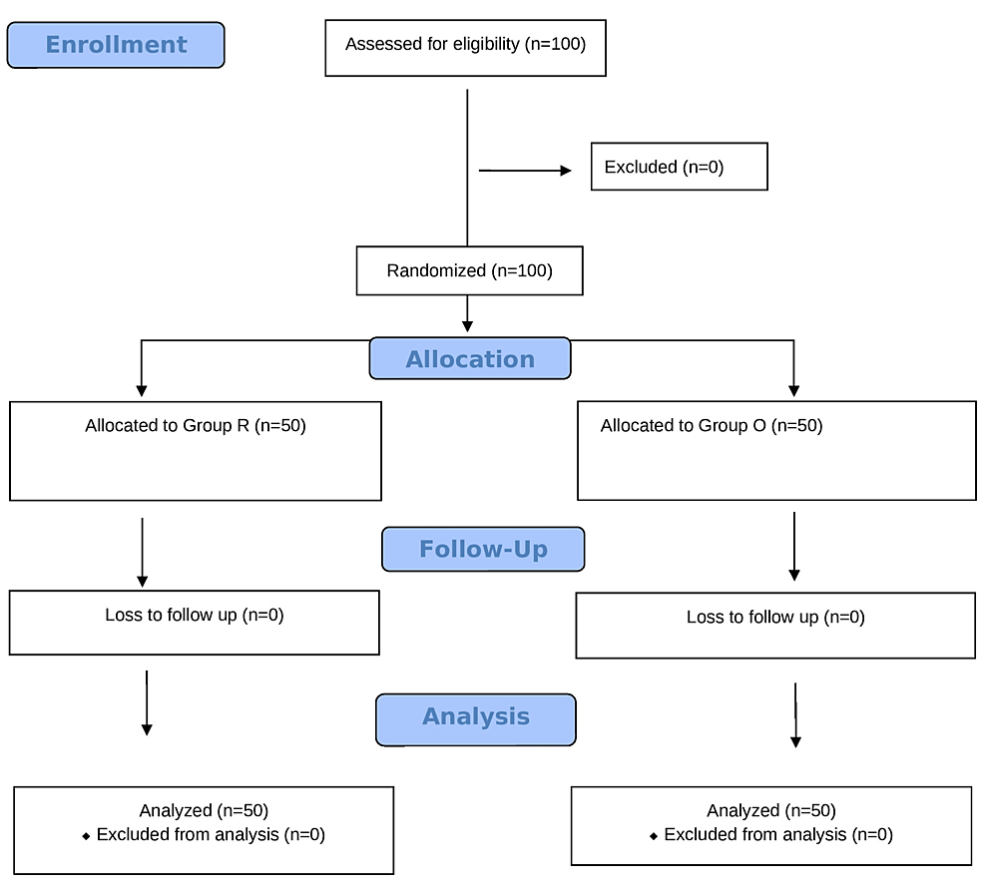
Consort flow diagram

This prospective, double-blind, randomised control trial was carried out in the Department of Anaesthesiology, Lady Hardinge Medical College, New Delhi, after approval by the ethical committee and clinical trial registry (CTRI/2018/05/014278) from November 2013 to March 2015. After obtaining informed written consent, we analysed 100 ASA grade I & II patients (Figure 1) in the age group of 18-60 years of female patients undergoing laparoscopic gynaecological procedures under general anaesthesia. We excluded the patient having a history of allergy to any drugs, a history of antiemetic medication in the last 24 hours before surgery, and those who refused to consent. A computer-generated random table was used for randomisation into two equal groups: Group R (ramosetron group) - i.v. Ramosetron 0.3mg given 30 minutes before the end of surgery and Group O (Ondansetron group) - i.v. Ondansetron 8mg is given 30 minutes before the end of surgery. The drug was prepared in equal volume (4 mL) and coded by one medical personnel who was not involved with the study or in data collection. The anaesthesiologist who was administering anaesthesia and doing data collection was blinded to the study drug.

After pre-anaesthetic checkups and written informed consent, the patients were familiarised with the technique of evaluation of PONV using a numeric scoring system. In the preoperative room, intravenous access was secured, and baseline vital signs were recorded. On arrival in the operation theatre, standard ASA monitors were attached, and each patient was pre-oxygenated for 3 minutes. Anaesthesia was induced with an injection of fentanyl 2 μg/kg, followed by an injection of thiopentone sodium in a dose sufficient to abolish the eyelash reflexes (4-6 mg/kg titrated dose), followed by 1 mg/kg of rocuronium bromide to provide neuromuscular blockade. The patient's lungs were ventilated with nitrous oxide, oxygen (50:50), and sevoflurane (1%-2%) with the help of a face mask for the next 90 sec. After that, the laryngoscopy was performed, and endotracheal intubation was done with a cuffed endotracheal tube of appropriate size. Standard ASA monitoring of vital parameters was done continuously till the completion of the surgery. Intraoperatively anaesthesia was maintained with sevoflurane and 50% nitrous oxide in oxygen, and a minimum alveolar concentration of 1.0-1.2 was maintained throughout the surgery. The study drug was given half an hour before the end of surgery. The residual neuromuscular blockade was antagonized with neostigmine (0.05mg/kg) and glycopyrrolate (0.01mg/kg) intravenously. Patients were extubated and shifted to the recovery room for further observation. Injection diclofenac and intravenous paracetamol (1g) 8hrly were used for postoperative pain.

Definition of relevant outcome measures

Nausea is defined as subjectively having an unpleasant feeling associated with being sick or awareness of the urge to vomit. Vomiting is defined as a forceful expulsion of gastric contents. Retching is laboured spasmodic, rhythmic contraction of the respiratory muscles without expelling gastric contents. PONV includes both nausea and vomiting.

Outcome

The primary objective of this study was to compare the effect of a single dose of ramosetron (0.3mg) with a single dose of ondansetron (8mg) for the prevention of PONV after general anaesthesia in laparoscopic surgeries. The secondary goals were to record the time of occurrence of the first episode of PONV, the need for rescue antiemetics, patient satisfaction scores, and to look for any side effects, e.g., headache, dizziness, sedation. Primarily we recorded the number of episodes of nausea and vomiting postoperatively between the intervals of 0-2hrs, 2-6hrs, 6-12hrs and 12-24hrs. The time of occurrence of the first episode of nausea and vomiting was noted from the time of study drug was administered. Observations were sub-grouped into early (0-6hrs) and late (6-24hrs) postoperative periods.

PONV was evaluated by using a numeric scoring system [6]. No nausea or vomiting - 0, only nausea - 1, nausea with vomiting once - 2, and vomiting two or more times in 30 minutes - 3. Severe PONV is defined as a PONV score of 3 or persistent nausea (>2hrs) and was treated with a rescue antiemetic injection of Metoclopramide 10 mg. Retching was not considered a separate entity. Patients who reported retching were classified as having nausea.

Patient's satisfaction score with the study medication was assessed using a 5-point Likert scale 24 hours after the end of drug administration: 1 (very satisfied), 2 (quite satisfied), 3 (neither satisfied nor unsatisfied), 4 (somewhat unsatisfied), and 5 (very unsatisfied) [14]. Adverse events were recorded throughout the 24 hours of duration in the postoperative period.

Statistical analysis

Sample size calculation was based on the study by sung-hoon Kim et al. [15]. Considering the incidence of nausea with ondansetron (77%) while with ramosetron (60%), the difference was 17% with alpha = 0.05 and power = 80%. It comes up to 134 in each group, but due to the limited number of laparoscopic gynaecological procedures in our hospital, we considered a minimum sample of 50 patients in each group (a total of 100 patients). All data were collected and analysed using computer software statistical package for social science (SPSS) version 20 (IBM Corp, Armonk, NY). The quantitative study was done by using the unpaired Student t-test. The qualitative research was done by using chi-square test and Fisher test. Results were presented as mean ± SD or percentage. A p-value of <0.05 was considered significant.

## Results

We enrolled and analysed 50 patients in each group for this randomised, double-blinded interventional study. Once the patient had been recruited, there was no dropout from this study. There were no significant statistical differences in age, body mass index, preoperative risk factors, duration of anaesthesia and surgery, as well as the type of surgery (Table 1). Postoperative pain was taken care of by NSAID diclofenac and intravenous paracetamol boluses. The analgesic requirement was comparable between the two groups.

**Table 1:** Patient characteristics PONV - postoperative nausea and vomiting

Variables	Group O	Group R	P-value
Age	28.38 ± 6.26	27.10 ± 4.70	0.125
BMI	23.26 ± 2.58	23.19 ± 2.18	0.443
Duration of Anaesthesia	99.70±33.19	97.40 ±37.69	0.373
Duration of surgery	83.90±33.20	80.60±33.79	0.312
Preoperative risk factors			
H/O PONV or Motion sickness	8	7	0.390
Non-smoker	48	49	0.279
Use of postoperative opioids	0	0	-
Type of surgical/diagnostic procedures			
1° infertility	23	27	0.212
2° infertility	21	13	0.046
AUB	1	2	0.279
Ectopic pregnancy	2	3	0.323
Hydrosalpinx	1	2	0.279
Ovarian cyst	2	4	0.200
Ruptured ectopic	0	1	0.157

We did not find any statistical differences in side effects between the two groups (Table 2). The overall incidence of PONV in the early postoperative period (0-6hrs) was 51%, while in the late postoperative period (6-24hrs), it was 13%, which was statistically significant (p-value < 0.001). The requirement of rescue antiemetic was higher in group O than in group R but not statistically significant. The patients were also equally satisfied with antiemetic drugs. We did not find any statistical differences in side effects (Table 3) between the two groups.

**Table 2:** Variables observed in our study PONV - postoperative nausea and vomiting. N - number, % percentage of population

Variables	Group O N (%)	Group R N (%)	P-value
Comparison of time of the first episode of PON/POV			
0-2 hours	13 (26%)	12 (24%)	0.392
2-6 hours	12 (24%)	14 (28%)	0.293
Incidence of PONV			
0-6 hours	25 (50%)	26 (52%)	0.421
6-24 hours	7 (14%)	6 (12%)	0.383
0-24 hours	26 (52%)	27 (54%)	0.421
PONV incidence at different time intervals			
0 - 2 hours	13 (26%)	11 (22%)	0.32
2-6 hours	20 (40%)	20 (40%)	0.5
6-12 hours	7 (14%)	6 (12%)	0.383
12-24 hours	0	0	-
Incidence of Nausea and Vomiting in 24 hours			
Nausea alone	21 (42%)	16 (32%)	0.150
Vomiting	7 (14%)	11 (22%)	0.149
Complete treatment response in 24 hours	24 (48%)	23 (46%)	0.421
Requirement of rescue antiemetic			
0-2 hours	0	0	-
2-6 hours	6 (12%)	2 (4%)	0.070
6-12 hours	4 (8%)	2 (4%)	0.200
12-24 hours	0	0	-
Patient satisfaction score (Mean ±S.D.)			
0-2 hours	2.30±1.74	2.36±1.69	0.431
2-6 hours	2.88±1.87	2.92±1.85	0.457
6-12 hours	2.32±1.39	2.26±140	0.415
12-24 hours	1.86±1.09	1.92±1.05	0.390

**Table 3:** Distribution of side effects N - number, % percentage of population

Side effects	Group O N (%)	Group R N (%)	P-value
Headache	5 (10%)	4 (8%)	0.363
Vertigo	0 (0%)	1 (2%)	0.157
Dizziness	0 (0%)	0 (0%)	-
Arrhythmias	0 (0%)	0 (0%)	-
Constipation	0 (0%)	0 (0%)	-
Abdominal pain	0 (0%)	0 (0%)	-

## Discussion

We observed an incidence of PONV (53%) even after a prophylactic antiemetic dose during the first 24 hours after surgery. This was mainly contributed by patient factors like female gender, non-smoker and history of PONV or motion sickness, which was subsequently aggravated by anaesthetic and surgical factors: use of nitrous oxide, sevoflurane and laparoscopic surgery. To reduce the confounding factor, we standardise the anaesthetic technique.

Although evidence suggests that using nitrous oxide enhances the risk of PONV, a recent study also means it does not increase the chances of PONV [16]. Our study compared two antiemetic drugs, Ondansetron and ramosetron, in female patients undergoing laparoscopic surgeries. There were no significant differences in the prevention of PONV between the two groups. Still, we noticed that overall, the first episodes of PONV were significantly higher in the early (0-6 hours) compared to the late (6-24hrs) postoperative period.

In our study, the incidence of PONV during the early and late postoperative periods was also comparable between the two groups. Kim et al., Suh et al., and Ansari et al. also reported similar incidences of PONV after the administration of ramosetron [12,17,18]. In some other studies, the incidence of PONV was less than in our study, which was reported by Banerjee et al., Agarkar et al., Ryu et al., and Swaika et al., [13,14,19,20]. The above differences may be because of differences in patient selection, the type of surgery, and intraoperative use of opioids and nitrous oxide (N_2_O) for maintenance of anaesthesia.

In our study, the incidence of nausea alone in group O and group R was 42% and 32%, respectively. The incidence of vomiting in 24hrs postoperatively was 14% and 22% in group O and group R, respectively. We observed no significant differences between the groups. Kim et al. and Hahm TS et al. demonstrated similar findings [12,21].

We found no statistical difference between the severity of PONV scores in the two groups at different time intervals. Suh et al., Kim et al., Ryu et al., and Agarkar et al. also found no difference in the severity of nausea between the ondansetron and ramosetron groups. In contrast, Banerjee et al. found that the severity of PONV was more after the second hour in the case of ondansetron than ramosetron [12-14,17,19]. In contrast to our study, Banerjee et al. used ondansetron in the dose of 4mg compared to 8mg [13].

We found a complete treatment response, i.e., neither nausea nor vomiting in the 24hrs postoperative period was 48% in group O compared to 46% in group R. No statistically significant difference was observed between the two groups (p = 0.421). Our study demonstrated similar results as studied by Ansari et al. and Ryu et al. [18,19].

We did not get any difference between patient satisfaction scores. Similar to our study, Kim et al., Ansari et al., and Ryu et al. also found that overall satisfaction scores were comparable between these two antiemetcs [12,18,19]. In our study, none of the patients required any rescue antiemetic during 0-2 hrs. During the 2-6hrs postoperative period, six patients in group O and two patients in group R, while during 6-12hrs, four patients in group O and two in group R required rescue antiemetic. This was similar to other studies by Kim et al. and Ansari et al. [12,18].

In our study, the most common adverse effect in both groups was headache (12% of patients in group ondansetron compared to 10% of patients in group ramosetron). Only one patient in group R had complaints of vertigo. These were statistically nonsignificant. Other studies have also reported side effects like headache, dizziness, and drowsiness.

Our study has several limitations. First, our sample size was limited. Second, in detail, we did not observe individual contributing factors (pregnancy, sevoflurane, laparoscopic surgery) to PONV. Third, we had not followed the long-term effect of ramosetron in follow-up.

## Conclusions

Our study concludes that ramosetron is as effective as ondansetron in preventing PONV incidence, severity and patient satisfaction for PONV prophylaxis within 24hrs of the postoperative period. Further studies are needed to know the effectiveness of ramosetron over ondansetron with a more robust sample size.
